# Emergency Management of Pediatric Orbital Pencil Trauma Outside the Operating Room: A Case Report

**DOI:** 10.7759/cureus.87645

**Published:** 2025-07-10

**Authors:** Anshu Lakra, Saurabh Trivedi, Ankita, Jitendra Kushwaha, Pooja Thaware, Md Yunus

**Affiliations:** 1 Trauma and Emergency Medicine, All India Institute of Medical Sciences (AIIMS) Bhopal, Bhopal, IND; 2 Anaesthesiology, All India Institute of Medical Sciences (AIIMS) Bhopal, Bhopal, IND

**Keywords:** non-operating room anesthesia, nora, orbital injury, pediatric trauma, penetrating foreign body, remote location anesthesia

## Abstract

Penetrating orbital injuries in pediatric patients are rare but potentially vision- and life-threatening emergencies. We present the case of a three-year-old girl who arrived at the emergency department with a penetrating orbital pencil injury. The foreign body was successfully removed under general anesthesia in a minor procedure room, following established non-operating room anesthesia (NORA) safety protocols. This case highlights the critical importance of rapid interdisciplinary coordination, meticulous preoperative assessment, and safe anesthetic conduct in non-traditional environments. Implementation of NORA principles in emergency trauma care can ensure timely intervention while maintaining perioperative safety standards in resource-limited or high-demand settings.

## Introduction

Orbital trauma involving foreign bodies demands urgent attention due to the risk of optic nerve damage, infection, and intracranial complications. In pediatric patients, such injuries pose additional challenges due to emotional distress, increased parasympathetic tone, anatomical delicacy, and limited cooperation. Penetrating orbital trauma requires timely diagnosis and an individualized anesthetic plan to prevent complications. Previous studies suggest that intraorbital foreign bodies near the optic nerve or globe should be managed with great caution due to risks of vision loss, hemorrhage, or intracranial extension [1,2].

Non-operating room anesthesia (NORA), although widely implemented in radiology and endoscopy, is infrequently used for trauma surgery, especially in emergency cases. However, the growing reliance on NORA across hospitals has shown promise in optimizing operating room efficiency and enhancing patient safety when access to the main operating theater (OT) is limited [3-5]. The updated guidelines from the European Society of Anaesthesiology further emphasize the importance of standardized procedural sedation practices in adults, reinforcing their relevance to emergency and non-OT settings [6]. This case illustrates the successful emergency management of a penetrating orbital foreign body in a pediatric patient. The procedure was performed under general anesthesia in a non-operating room setting, adhering to established NORA protocols.

## Case presentation

**Figure 1:**
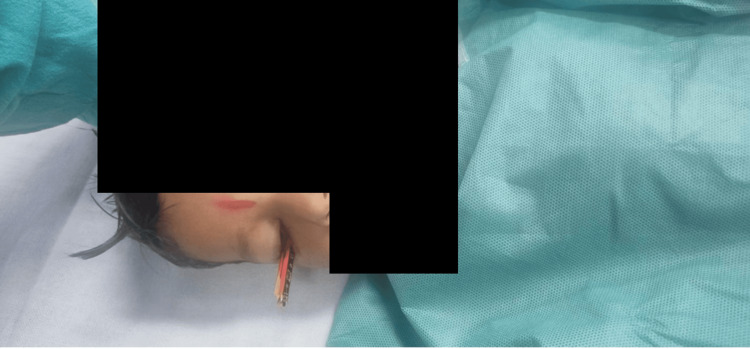
Patient with a pencil lodged in the right medial orbital region.

A three-year-old girl presented to the emergency department with a pencil penetrating her right orbit while playing at an Anganwadi school. The pencil was visibly lodged at the medial orbital margin (Figure 1). Upon arrival, the child was conscious, oriented, and hemodynamically stable. She was kept nil per os (NPO) in anticipation of potential anesthetic intervention for foreign body removal.

An urgent ophthalmologic evaluation raised suspicion of orbital bone involvement. A non-contrast computed tomography (NCCT) scan of the brain and orbit was immediately performed, which revealed a hyperdense foreign body in the right extraconal space. The object had breached the lateral orbital wall and extended up to the superior orbital fissure, displacing both the optic nerve and the posterior globe, without any evidence of globe rupture. The decision was made to proceed under NORA conditions in the minor OT. All safety checks were performed, including verification of oxygen availability, suction, resuscitation equipment, monitors, and the presence of trained personnel, in accordance with ASA and NORA guidelines.

The patient was transferred to the minor OT after ensuring full compliance with NORA guidelines. Standard ASA monitors were attached, including ECG, pulse oximetry, non-invasive blood pressure, and capnography. Prior to induction, a comprehensive NORA safety checklist was completed. A fully equipped pediatric resuscitation cart was confirmed to be available in the minor OT, containing airway adjuncts, emergency medications, and a defibrillator. 

Given the orbital location of the penetrating foreign body, the team anticipated the possibility of an oculocardiac reflex (OCR) during manipulation. Continuous ECG monitoring was employed to detect bradyarrhythmias, and intravenous glycopyrrolate was administered as a preemptive anticholinergic to attenuate vagal responses. These precautions were essential to maintaining hemodynamic stability throughout the procedure.

**Figure 2:**
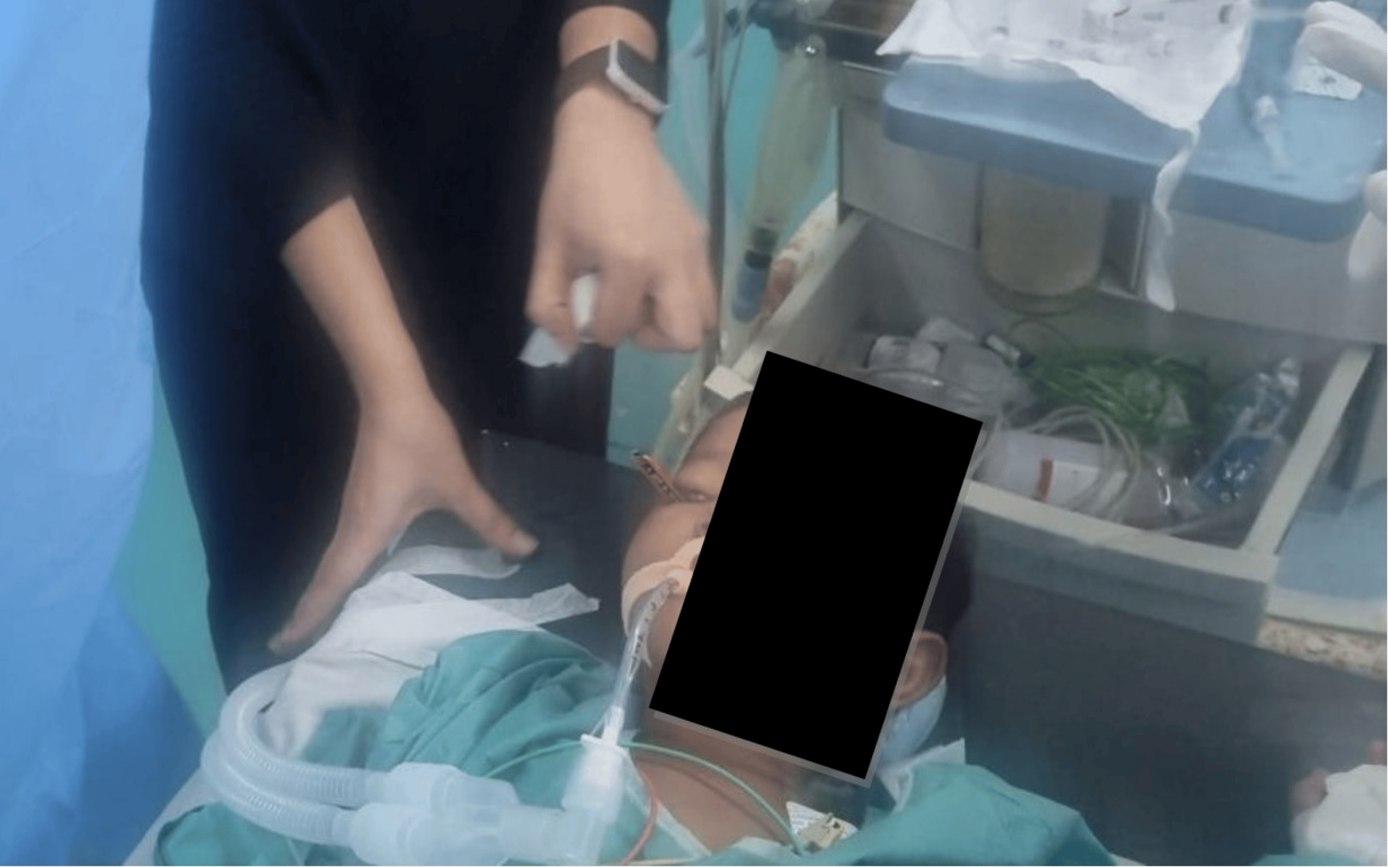
Intraoperative management under general anesthesia in NORA settings. The child is seen under general anesthesia with endotracheal intubation and standard ASA monitors attached. The procedure was conducted in a minor operating room following NORA safety protocols. A multidisciplinary team prepared for controlled removal of the penetrating orbital pencil. NORA: non-operating room anesthesia.

Premedication included intravenous fentanyl 20 mcg (1 mcg/kg) and glycopyrrolate 0.15 mg (0.0075 mg/kg). Anesthesia was induced using intravenous propofol 40 mg (2 mg/kg) and atracurium 7.5 mg (0.375 mg/kg). Endotracheal intubation was performed with minimal head manipulation to avoid increased intraorbital pressure or displacement of the foreign body (Figure 2). Anesthesia was maintained with a mixture of oxygen and air, supplemented with sevoflurane. Intravenous paracetamol 300 mg (15 mg/kg) and ondansetron 2 mg (0.1 mg/kg) were administered for analgesia and antiemetic prophylaxis, respectively. 

**Figure 3:**
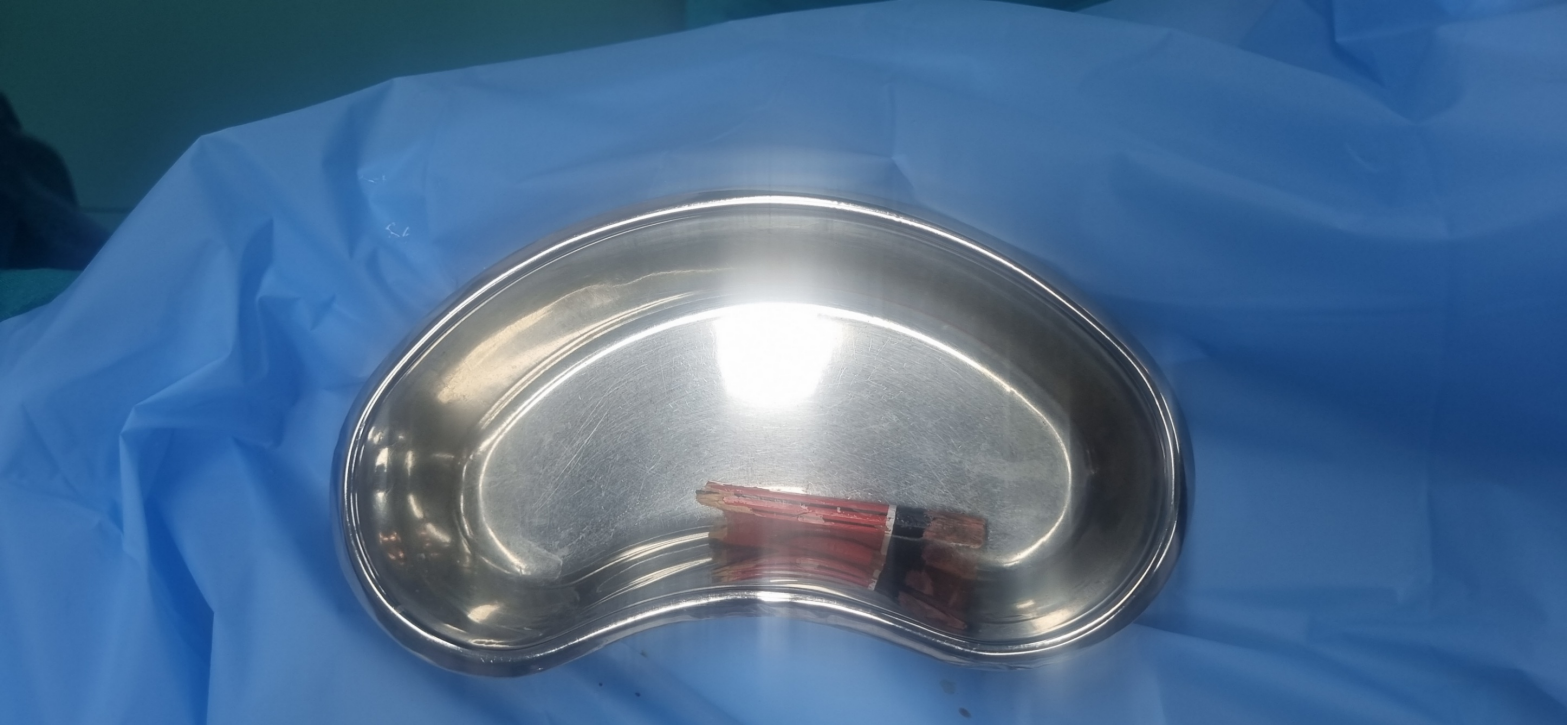
Extracted foreign body. The wooden pencil removed from the medial orbital region is displayed post-extraction. It clearly shows the dimensions and structure of the pencil, highlighting its sharp tip and length, which reflect the potential depth of penetration and risk to the orbital structure.

**Figure 4:**
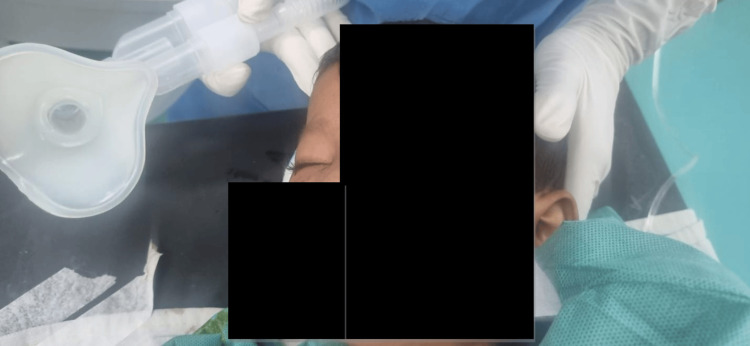
Postoperative recovery after foreign body removal. The patient is seen in the immediate postoperative period under observation following successful removal of the orbital pencil. She is breathing spontaneously. No signs of ocular hemorrhage or neurological compromise were noted.

The neurosurgical team extracted the foreign body, followed by wound exploration and closure by the ophthalmology team (Figure 3). The total surgical duration was approximately 45 minutes, and the procedure proceeded without any intraoperative complications. To minimize coughing and bucking during emergence, we maintained an adequate depth of anesthesia with sevoflurane until the end of the procedure. Intravenous lignocaine (1.5 mg/kg) was administered 2-3 minutes prior to extubation to blunt airway reflexes. The patient was extubated in a deep plane of anesthesia with spontaneous ventilation in the left lateral position, after confirming adequate suppression of airway reflexes and hemodynamic stability. Noxious stimuli were avoided during emergence to ensure a smooth and complication-free extubation. The patient was extubated uneventfully and transferred to the high-dependency unit (HDU) for postoperative monitoring (Figure 4). She was discharged in stable condition on postoperative day 3.

## Discussion

This case underscores the safe and effective application of general anesthesia in a NORA setting for the management of emergency orbital trauma in a pediatric patient. Traditionally, NORA has been associated with radiological, gastrointestinal, and dental procedures; however, its utility is increasingly recognized in trauma management, particularly in resource-limited environments where immediate access to a fully equipped OT may be restricted [3-5].

Adherence to established NORA protocols, including thorough equipment checks, continuous patient monitoring, and the presence of trained personnel, was essential to the favorable outcome in this case. The importance of standard safety measures, such as pre-induction readiness, was amplified due to the anatomical proximity of the foreign body to critical orbital structures, including the optic nerve and globe [1,2].

NORA environments inherently pose challenges such as restricted workspace, unfamiliar equipment layout, and limited availability of emergency backup. These factors demand heightened vigilance, meticulous preoperative planning, and strong interdisciplinary coordination. In this case, early involvement of ophthalmology, neurosurgery, and anesthesiology ensured a structured approach, minimizing procedural delays and potential complications.

Airway management was particularly critical, as any undue pressure or movement could have worsened orbital injury or increased intraocular pressure (IOP). A smooth emergence from anesthesia was successfully achieved, reducing the risk of IOP-related exacerbation of injury or hemorrhage. This is consistent with prior findings by Cravero et al., who emphasized the importance of monitoring during pediatric sedation outside the OR to mitigate complications [7]. Similarly, Urman et al. have highlighted the role of structured protocols in improving safety outcomes in NORA cases [8].

Furthermore, recent literature has explored expanding the scope of NORA in pediatric populations. A review by Erondu and Mahoney also supported the use of general anesthesia in NORA environments for pediatric ophthalmic interventions when access to a formal OT is not immediately available [9].

Overall, this case contributes to the growing body of evidence supporting the role of NORA in emergency pediatric trauma care. It demonstrates that with appropriate planning and adherence to safety guidelines, life- and vision-threatening emergencies can be managed effectively outside of the conventional operating room.

## Conclusions

This case underscores the feasibility and safety of performing urgent pediatric orbital foreign body removal under NORA in a resource-limited, high-demand setting. Despite the lack of immediate access to a conventional operating room, the procedure was successfully executed with strict adherence to NORA protocols, multidisciplinary collaboration, and anticipation of potential complications such as OCR and malignant hyperthermia.

What sets this case apart is the complex anatomical location of the foreign body, extending to the superior orbital fissure and displacing the optic nerve and posterior globe, managed effectively in a non-traditional surgical environment. This case highlights how structured perioperative planning, institutional readiness, and clinical vigilance can enable safe anesthesia and surgical intervention outside the operating room, even in high-risk pediatric trauma scenarios. Our experience contributes to the growing evidence supporting the utility of NORA in emergency pediatric care and may serve as a practical reference for centers operating under similar logistical constraints.
